# Case of a Fistula in Ano, Cured by Means of a Caustic

**Published:** 1784

**Authors:** 


III. *Cafe of a Fiftula in Ano, cured by means of a Cauftic. Communicated in a letter to Dr.* Simmons, *by Mr.* P. Dillon, *late Surgeon of the* 105*th Regiment of Foot.*

A SOLDIER's wife, belonging to the late 105th regiment, applied to me on account of a difcharge, which had originated in the laft month of pregnancy, and which, through motives of delicacy, fhe had kept to herfelf, for the fpace of feven months, till her health health was ſo affected, that ſhe thought herſelf in danger of dying. She informed me, that large quantities of matter came away with her ſtools; and on examination, I found three large openings on the right ſide of the anus, about an inch from the ſphincter, which diſcharged a great deal of matter; a ſmall quantity of pus was alſo to be ſeen about the extremity of the rectum, which convinced me that the three openings communicated with the inteſtine.—On introducing a probe into the central opening, I found that it reached a conſiderable way; but no communication with the rectum could be perceived by the whole length of my fore finger; though on injecting milk and water into the abſceſs, I found that it came out at the anus. I alſo found that the two lateral openings tended towards the central one; and on introducing three probes, one into each opening, I found that they communicated with each other, about two inches and a half below the ſurface, in a right line with the rectum.

In making an inciſion to this depth, ſuch an hæmorrhage enſued, as induced ſyncope; but in about ten minutes after the bleeding was ſtopped, the patient's breathing and pulſe began to return. After dreſſing the wound, I

 gave gave her a saline draught, with a few drops of Thebaic Tincture, and she rested well that night. In two days after the operation, I opened the wound, and found the anterior abscess in a state of inflammation, and going on as well as I could expect, no discharge coming from the posterior abscess, which I think proceeded from the general inflammation excited in the parts.

Previous to the operation, an emollient clyster had been administered; and after the operation, the patient continued to be pretty free in her body, took the bark largely, and went on well, till about the eighth day, when the discharge began to be profuse again from the posterior abscess.

I now introduced a pessary into the rectum, and found the communication between it and the posterior abscess about two inches deeper than the posterior part of the wound already made. The apprehension of wounding a second time any considerable branches of an artery, deterred me from making any farther use of the knife; but as the poor woman was sinking under the violence of the discharge, I determined to attempt a cure, by destroying the texture of the part. For this purpose, I introduced duced a cauſtic bougie into the wound, and ſuffered it to remain there an hour, during which time the patient was in violent pain. On withdrawing it, I was in hopes that enough, though not more than I wiſhed, of the parts was deſtroyed. The wound was dreſſed lightly, and an inflammation enſued in the abdomen, attended with ſlight ſpaſms; but theſe were leſs violent than I expected, and were removed by a few anodyne draughts.

At the end of three days, an inflammation appeared round the edges of the ſlough, which ſeparated entirely in ſeven or eight days after, and granulations began to form from the edges towards the center of the wound, which, in the courſe of twenty days, entirely filled it up. The external openings remained longer than I could have expected; but the patient perſevering in the uſe of the bark, very ſoon began to recover her ſtrength, and in the courſe of ſix weeks the wound was completely healed.— She continues well, and ſeems to be in no danger of a return of her complaint.

The ſucceſs that attended this method of cure in the above caſe, and the probability there is of its ſucceeding in other caſes of the ſame kind, which have hitherto been deemed incurable, and under which we have ſeen ſo many patients ſink, will, I hope, recommend it to the notice of practitioners. I think, that in ſuch caſes, the Surgeon ſhould never carry his knife ſo far, as to riſque either the patient's life or his own character; and that in deeply-ſeated fiſtulæ (and ſuch only) the cure by cauſtic may be attempted with a much greater chance of ſucceſs.

*Edinburgh,*
*September* 14, 1784.

